# A FEN 1-driven DNA walker-like reaction coupling with magnetic bead-based separation for specific SNP detection

**DOI:** 10.3389/fbioe.2023.1279473

**Published:** 2023-11-03

**Authors:** Shijie Xu, Jian Chen, Fang Yang, Zhihao Yang, Jianrong Xu, Lanyue Wang, Lina Bian, Lihua Liu, Xiaoyu Zhao, Yunshan Zhang

**Affiliations:** ^1^ School of Chemistry and Chemical Engineering, Hunan University of Science and Technology, Xiangtan, Hunan, China; ^2^ Research Center for Intelligent Sensing Systems, Zhejiang Laboratory, Hangzhou, China; ^3^ School of Electronic Engineering, Nanjing Normal University, Taizhou College, Taizhou, China; ^4^ College of Materials and Environmental Engineering, Hangzhou Dianzi University, Hangzhou, China

**Keywords:** single-nucleotide polymorphism, flap endonuclease 1, DNA walker-like reaction, streptavidin magnetic beads, KRAS gene

## Abstract

Single-nucleotide polymorphism (SNP) plays a key role in the carcinogenesis of the human genome, and understanding the intrinsic relationship between individual genetic variations and carcinogenesis lies heavily in the establishment of a precise and sensitive SNP detection platform. Given this, a powerful and reliable SNP detection platform is proposed by a flap endonuclease 1 (FEN 1)-driven DNA walker-like reaction coupling with a magnetic bead (MB)-based separation. A carboxyfluorescein (FAM)-labeled downstream probe (DP) was decorated on a streptavidin magnetic bead (SMB). The target DNA, as a walker strand, was captured by hybridization with DP and an upstream probe (UP) to form a three-base overlapping structure and execute the walking function on the surface of SMB. FEN 1 was employed to specifically recognize the three-base overlapping structure and cut the 5′flap at the SNP site to report the walking event and signal amplification. Considering the fact that the fluorescence was labeled on the cleavage and uncleavage sequences of DP and the target DNA-triggered walking event was undistinguishable from the mixtures, magnetic separation came in handy for cleavage probe (CP) isolation and discrimination of the amplified signal from the background signal. In comparison with the conventional DNA walker reaction, this strategy was coupling with SMB-based separation, thus promising a powerful and reliable method for SNP detection and signal amplification.

## 1 Introduction

SNP is involved in the polymorphism of single nucleotide variation at the genetic level in different biological organisms, which is in turn associated with genetically transmitted traits of variation in the genome (Kashif et al., 2021; Choi et al., 2022). When an SNP is located in the coding region of a specific gene, it can directly affect the structural and functional changes of proteins and induce mutation-related diseases (Gerasimavicius et al., 2022). However, if the SNP is located in the non-coding regions of the genome, the mutation-related disease is difficult to identify and may be severed as a genetic marker in population genetics (Toropainen et al., 2022). Regardless of how the mutation is generated, it is a powerful predictor and biomarker for the prediction, diagnosis, and treatment of a variety of diseases (Breiteneder et al., 2020; Battaglin et al., 2021). KRAS-mutated oncogenes are reported to be more likely to develop cancer-related diseases, and more than 11% of all cancers are defined as a result of mutation in the KRAS gene, which is a growing health concern (Huang et al., 2021). For example, pancreatic cancer, with a 5-year survival rate of less than 5%, is one of the most lethal cancers caused by a mutation of the KRAS gene with rapid development and a poor prognosis (Suzuki et al., 2022). Notably, if patients miss opportunities for early diagnosis, they have a median survival of only 2–15 months (Yokose et al., 2020; Zhang et al., 2023b). Therefore, early detection of KRAS gene SNPs is an effective approach for prompt intervention and treatment. Meanwhile, mutation plays an important role in the development of cancer-related diseases, thus leading to the creation of potential targetable vulnerabilities (Wang et al., 2022b; Deng et al., 2022; Lv et al., 2023). Many efforts are also focused on elucidating the basis of disease-associated genes by detecting the important associated single-base mutation. The association between gene-related diseases and SNPs can help us better understand the pathogenesis of diseases and attract the interest of contemporary scientists (Xiang et al., 2021; Yi et al., 2022; Wang et al., 2023). Therefore, it is imperative to develop a detection platform for SNPs, especially for genetic diseases. In recent years, various genotyping techniques have been developed for SNP detection, such as Sanger sequencing, the Taqman probe, amplification-refractory mutation system PCR, and molecular beacon probes (Kalendar et al., 2021; He et al., 2023). These techniques provide high accuracy in detection performance. However, these methods usually require tedious operation and expensive supporting instruments in the detection process, which cannot guarantee detection sensitivity, specificity, or stability.

To address these challenges, many efforts have been made to develop simple and rapid SNP detection assays based on the specific recognition and signal amplification of enzymes (Ahmed et al., 2022). For example, FEN 1, a structure-specific endonuclease, plays a critical role in maintaining genetic integrity by participating in DNA replication, recombination, and repair (Yang et al., 2022). FEN 1 can recognize the three-base overlapping structure formed by the target, upstream probe (UP), and downstream probe (DP), and accurately cut the 5′overhanging flap of the DP. Thus, FEN 1 has a natural advantage in detecting single-base mutations. Importantly, this structure-specific endonuclease also has the advantage of structure-based selection and cleavage of unique DNA substrates with remarkable efficiency outside of cells. In other words, its main action is to precisely recognize the three-base overlapping structure and remove the 5′flap-stranded overhang of branched duplex DNA both *in vivo* and *in vitro* (Zhu et al., 2023). Precise selectivity of the incision site based on FEN 1 provides a useful strategy for site-specific SNP detection. Taking advantage of the special properties of FEN 1, a series of FEN 1-based DNA or RNA detection biosensors have been developed. For instance, Wang et al. developed a FEN 1-assisted swing arm DNA walker for electrochemical detection of ctDNA, which could facilitate target recycling cascade amplification and showed a detection limit of 0.33 fM (Wang et al., 2022a). Wei et al. used Taq DNA ligase to cut the 5′flap of circular DNA substrate in the presence of FEN 1 and quantitatively detected the activity of FEN 1 in human cancer cells with SYBR Green I as an indicator (Wei et al., 2022). Although FEN 1 has made great progress in nucleic acid detection, the characteristics and advantages of FEN 1 in recognizing single-base mutations are still underutilized in SNP detection. However, there are still several issues that need to be considered before designing SNP biosensing based on FEN 1. To form the three-base overlapping structure, the target DNA dispersed in an aqueous solution requires hybridization with UP and DP by free diffusion, and low hybridization efficiency has been observed so far owing to the low collision rates between probes (Tang et al., 2021b). Thus, the three-base overlapping structure is difficult to recruit. Correspondingly, the three-base overlapping structure formed in the diluted solution has on potent driver for the invasive reaction owing to unconcentrated cleavage sites.

Alternative approaches still need to be explored to promote structure-specific substrate recruitment and precise cleavage site enrichment so as to accelerate the formation of three-base overlapping structures and trigger an invasive reaction (Han et al., 2023). Meanwhile, there are many efforts to seek solutions to these limitations, such as probe-decorated microspheres and magnetic separation. Specifically, the probes can be modified on microspheres by covalent conjugation or specific adsorption, and the target DNA can therefore be enriched on microspheres via the hybridization reaction (Ren et al., 2022). Moreover, the local high concentration of probes on microbeads also benefits cleavage site enrichment. In addition, magnetic bead separation also seems to be a good option for capturing and enriching target DNA from the mixtures in a few minutes. This is emerging as a promising alternative to substrate recruitment. For example, Balakrishnan et al. employed superparamagnetic bead particles to realize the separation of ctDNA on a microfluidic platform, thereby enabling early cancer detection (Balakrishnan et al., 2021). Tang et al. designed an HIV DNA biosensor by combining upconversion luminescent probes and magnetic beads to realize low background interference detection (Tang et al., 2021a). Yuan et al. established a multifunctional magnetic nanoparticle probe coupling with a hybridization chain reaction platform for microRNA detection (Yuan et al., 2017). Compared with monodisperse probes, the magnetic nanoparticle-decorated probe can not only enhance its local concentration but also offer a potential driving force for the invasive response of FEN 1.

**SCHEME 1:**
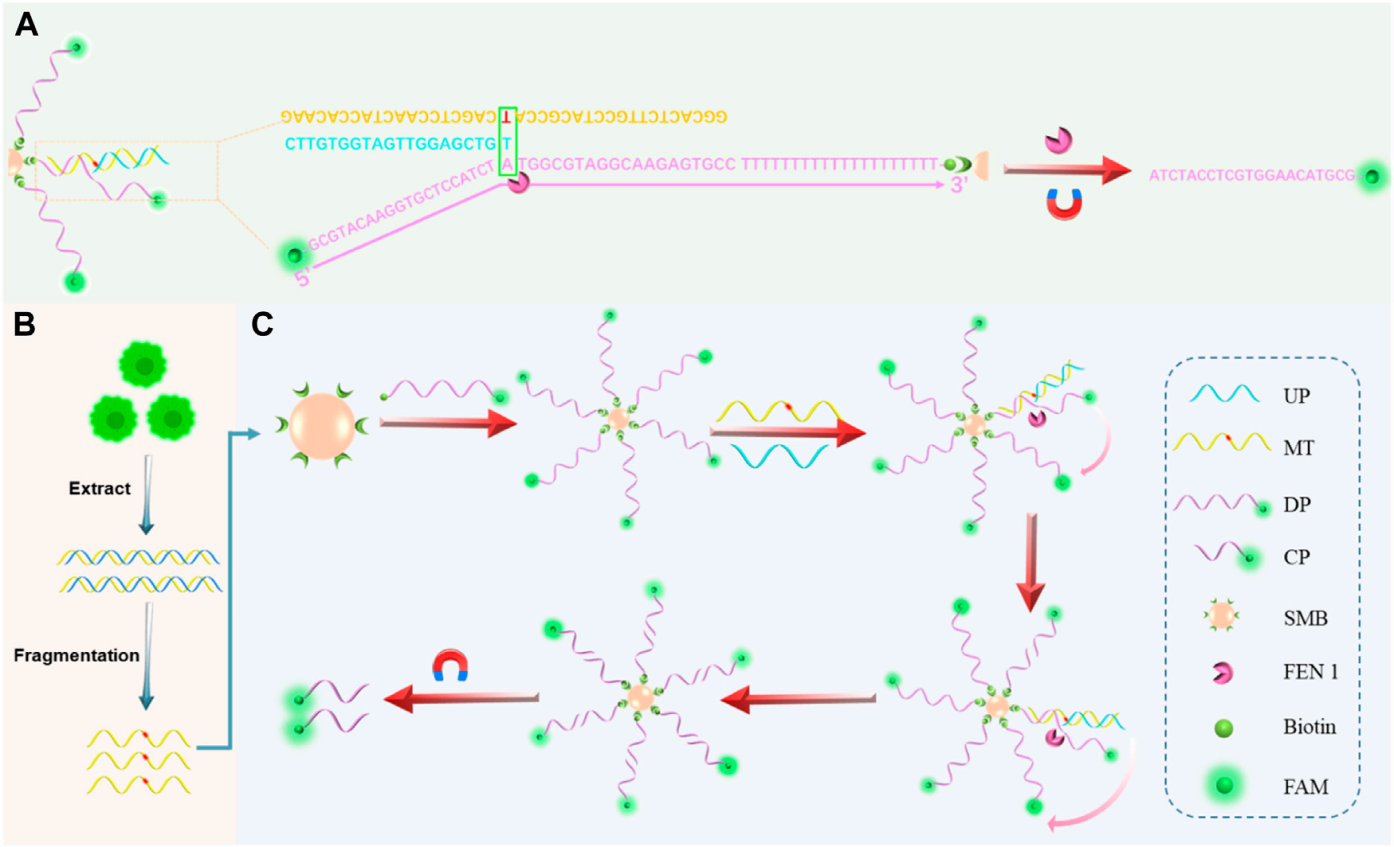
General schematic of the proposed assay. **(A)** Hybridization of target DNA, DP with sticky ends, and UP to form a three-base overlapping structure recognized by FEN 1. **(B)** Gene mutation sample extraction, fragmentation, and SNP detection processes in cells. **(C)** Schematic illustration of FEN 1-driven DNA walker-like reaction coupling with magnetic bead-based separation for specific SNP detection.

Given the aforementioned limitations and improvements, this work attempts to propose a target DNA captured by FEN 1-driven DNA walker-like reaction and 5′ flap cleavage to realize signal amplification and sensitive detection of SNP (Scheme 1). The FAM-labeled DP was modified and enriched on SMB by streptavidin-biotin conjugation to facilitate a DNA walker-like reaction. In the presence of target DNA, hybridization of the three-base overlapping structure was detected by FEN 1, and the 5’ flap was cut to generate signals. Owing to the specific structure-based selection and cleavage properties of FEN1, the DNA walker-like reaction was able to sustain walking around DP-decorated SMB to form a cycle cleavage reaction and generate a large amount of cleavage probe (CP). Meanwhile, the separation of CP from uncleaved sequences based on magnetic manipulation could effectively distinguish mutant target (MT) and wild target (WT) to avoid false positive signals. This strategy could provide a powerful and reliable method to effectively detect SNPs.

## 2 Materials and methods

### 2.1 Materials and equipment

FEN 1 was purchased from New England Biolabs, Inc. (Beijing, China). BeyoMagTM Streptavidin Magnetic Beads (SMB), 200 nm in size, were purchased from Beyotime Biotechnology Co. Ltd. (Shanghai, China). All DNA oligonucleotides listed in Supplementary Table S1 were custom-synthesized and purified by Sangon Biotech Co. Ltd. (Shanghai, China) with high-performance liquid chromatography. The 0.1% DEPC-treated deionized water (˃18 MΩ cm, Milli-Q, Millipore, Merck, Darmstadt, Germany) was used for all experiments.

### 2.2 Detection and signal amplification procedures

First, 1 μM of UP, 1 μM of DNA mimics (MT/WT), and 5 μM of biotin and FAM-modified DP were annealed at 95 °C for 5 min and then cooled to room temperature for further use. Second, 0.5 mg of SMB was washed with pure H_2_O two times and mixed with 1 μM FAM-modified DP in 100 μL pure H_2_O at 25°C for 1 h. Third, the prepared SMB/DP were collected by magnetic bead-based separation and dispersed in FEN 1 buffer for further use. The FEN 1-induced cleavage reaction was then performed in 1 × Thermopol reaction buffer (20 mM Tris-HCl, 10 mM (NH_4_)_2_SO_4_, 10 mM KCl, 2 mM MgSO_4_, 0.1% Triton^®^ X-100, pH 7.4 @ 25°C), containing 10 U FEN 1, 0.1 μM UP, target DNA, and the above SMB/DP in a total volume of 100 μL at 55°C for 5 h. Finally, the supernatant was collected by magnetic absorption and diluted to 200 μL with FEN 1 buffer for analysis by fluorescence spectrophotometer.

### 2.3 Gel electrophoresis analysis

To verify the viability of the enzyme digestion reaction, the reaction products were analyzed by 15% polyacrylamide gel electrophoresis (PAGE) in 1× TBE buffer (2 mM EDTA, 89 mM boric acid, 89 mM Tris, pH 8.0) at 80 V constant voltage for 60 min at room temperature. Gel imaging was performed using the Bio-Rad PowerPac Basic Power (Bio-Rad Laboratories, Segrate, Italy). The gels were then pulled out, stained in ultra-Gel-Red dye (Vazyme Biotech Co., Ltd., Nanjing, China) for 30 min, and imaged with the Fluorescence&Chemiluminescence Gel Imaging System (Peiqing Science and Technology Co., Ltd., Shanghai, China).

### 2.4 Fluorescent spectroscopy analysis

Fluorescence spectra were recorded using a PerkinElmer spectrofluorometer (FL 6500) with both emission and excitation slits of 10 nm. The excitation wavelength was 490 nm, and the emission wavelength ranged from 510 to 570 nm at a voltage of 550 V. The fluorescence peak at an emission wavelength of 518 nm was used to evaluate the signal intensity.

### 2.5 Cell culture and genomic DNA extraction

The human pancreatic carcinoma cell line (PANC-1 cells), human cervical carcinoma cell line (HeLa cells), human lung adenocarcinoma cell line (A549 cells), and human embryonic kidney cell line were selected to check the detection performance of the SNP biosensor and were purchased from Beyotime (Shanghai, China). These cells were cultured in Dulbecco’s modified Eagle medium (DMEM, Biosharp, China) containing 1% penicillin-streptomycin (Invitrogen) and 10% fetal bovine serum (FBS, Biosharp, China) at 37°C in a humidified atmosphere. Total DNA was isolated from cultured cells using the MolPure^®^ cell/tissue DNA kit (Yeasen Biotechnology, Shanghai, China) according to the manufacturer’s protocol with minor modifications. DNA concentration and quality were measured using a Nanodrop ONE spectrophotometer (Thermo Fisher Scientific, Waltham, United States), and the biosensor constructed during this study was used to monitor SNP in biological samples.

## 3 Results and discussions

### 3.1 Design and principle of the biosensor

The principle of the proposed biosensor for the detection of KRAS gene mutations is depicted in Scheme 1. The biosensor consisted of cyclic amplification of the target signal formed by FEN 1-driven DNA walker-like reactions and SMB-based separation. For the mechanism of the FEN1-driven DNA walker-like reaction, DP modified with biotin at one end and fluorescent dye FAM at the other end was first used as the track strands. Then, the biotinylated DP was fixed and enriched on SMB through biotin-streptavidin interaction to recruit the target DNA. The UP, as a capture probe, contained a partial complementary sequence to the target DNA. In the presence of MT, UP captured MT by hybridization with its partially complementary sequence, and the captured MT continued to hybridize with DP on the surface of SMB to form a three-base overlapping structure. Subsequently, FEN 1 specifically recognized the three-base overlapping structure, and the FAM-labeled 5′-flap of DP was cleaved and released from SMB. The cleaved UP/MT hybridization was used as a traveling strand for autonomous movement along the dense DPs on the SMB surface, and it could continue to interact with another DP, thereby releasing abundant cleaved fluorophores and generating accumulated signals. Conversely, no three-base overlapping structure was formed when the WT was present. As a result, the 5′-flap of the DP was not cleaved by FEN 1, and the DNA walker-like reaction could not be initiated, resulting in only a weak background signal. The cleaved fluorescence (specific signal) generated by MT was mixed with uncleaved fluorescence that would interfere with the positive signal from the background signal. Through magnetic separation, the cleaved fluorescence can be differentiated from the mixed solution. Therefore, the combination of FEN 1-driven DNA walker-like reactions and SMB-based separation enables SNP site-specific detection, signal identification, and signal amplification.

### 3.2 Feasibility analysis

**FIGURE 1:**
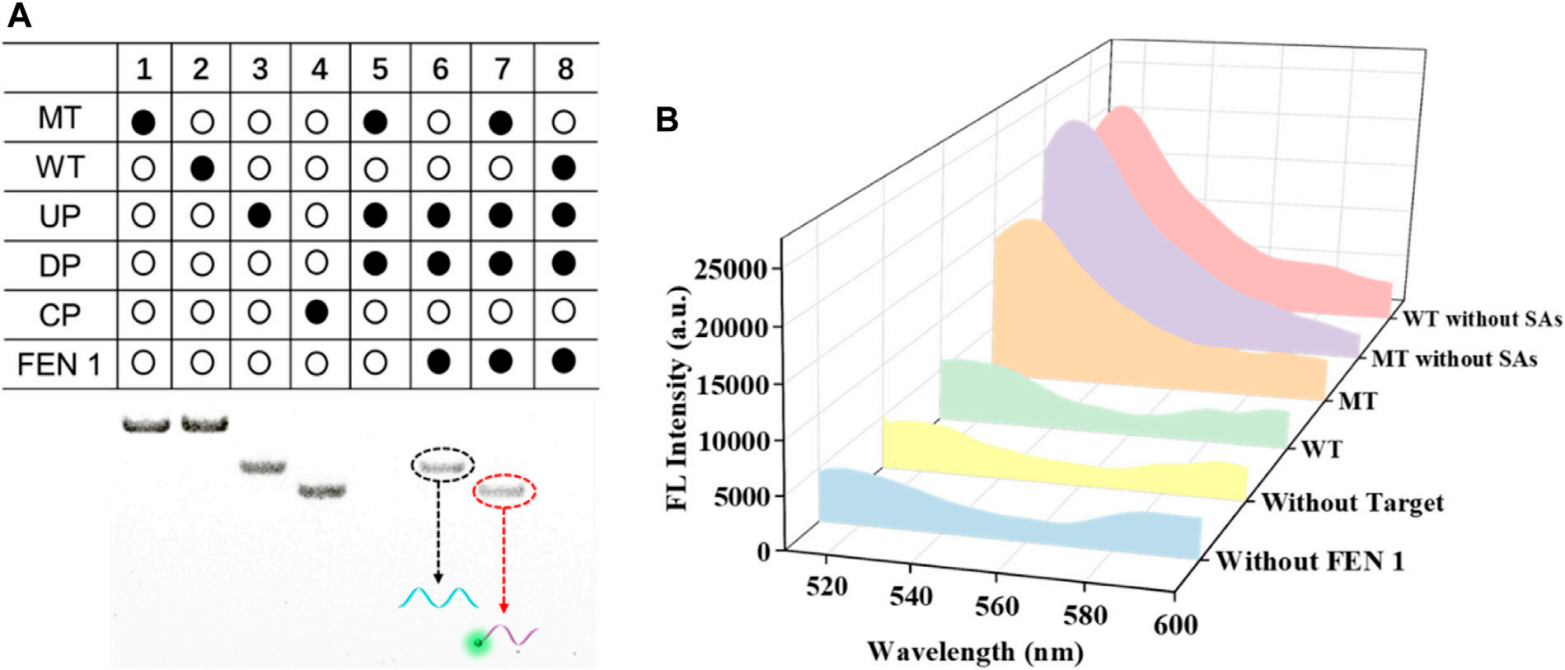
Feasibility analysis of this designed biosensor by PAGE **(A)** and fluorescence **(B)** experiments.

To test the feasibility of FEN 1-driven DNA walker-like reaction coupling with SMB-based separation for KRAS gene mutation detection, polyacrylamide gel electrophoresis and fluorescence spectroscopy were employed. As shown in Figure 1A, the components of MT, WT, UP, and DP were assigned to channels one to four, respectively. In the absence of FEN 1, no band was observed in the supernatant of channel 5 because the DNA walker-like reaction could only be driven by FEN 1 and these probes were partially complementary-pairing with excess DP and removed by magnetic separation. When MT was not present, only an UP band was observed in channel 6, indicating that the three-base overlap structure could not be formed by the loss of either component or that the DNA walker-like reaction failed to be driven. A clear CP band could be observed in channel 7 in the presence of MT, while no band appeared in channel 8 in the presence of WT. These results indicated that only MT could trigger the FEN 1-driven DNA walker-like reaction.

We also validated the feasibility of the proposed sensor using fluorescence spectroscopy again, and the results are shown in Figure 1B. In the absence of FEN 1, a negligible fluorescence signal was observed, indicating that no DNA walker-like reaction occurred. When no target DNA appeared, the fluorescence signal was weak. Similarly, WT led to a weak fluorescence signal, which could be attributed to the specific detection of the FEN 1-driven DNA walker-like reaction. On the contrary, depending on the MT present, a noticeable fluorescence signal was observed. In addition, an obvious fluorescence signal appeared from either MT or WT without the aid of magnetic separation due to the mixture of cleaved and uncleaved CP. These results suggest that there are reasons to believe that magnetic separation plays a key role in positive signal discrimination from the background signal. The above experimental results support the principle of this proposed biosensor based on FEN 1-driven DNA walker-like reaction coupling with magnetic bead-based separation for accurate SNP detection.

### 3.3 Optimization of detection conditions

**FIGURE 2:**
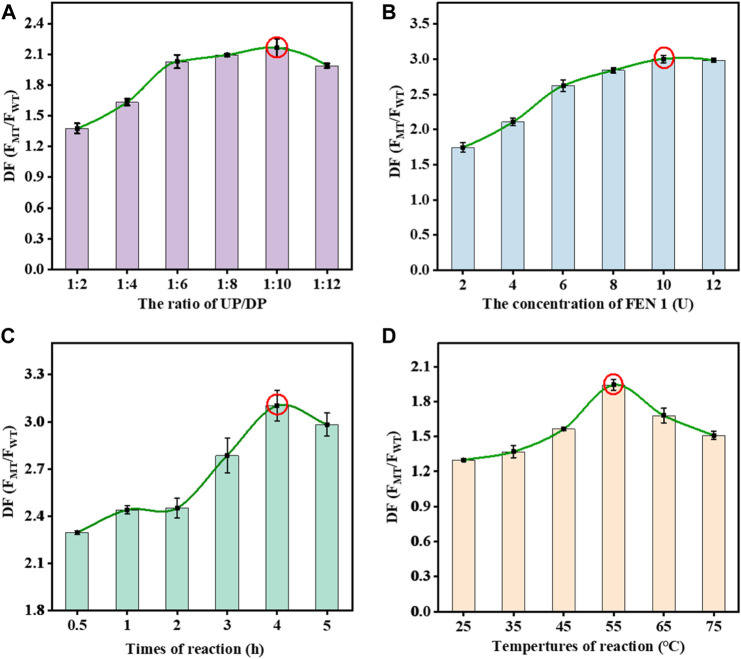
Effects of the UP/DP ratio **(A)**, FEN 1 concentration **(B)**, reaction time **(C)**, and reaction temperature **(D)** on the response of the proposed biosensor. Error bars: SD, *n* = 3.

It is essential to establish a correlation between signal response and experimental parameters to obtain optimal SNP detection performance. The ratio of fluorescence intensity between MT and WT was used to reflect the optimal conditions. The formula was defined as the following: DF = FMT/FWT, where FMT was the fluorescence intensity of MT and FWT was the fluorescence intensity of WT. Taking advantage of the fixed DP in SMB, we optimized the UP/DP concentration ratio with the maximum DF value. With the rise of DP concentration in the mixtures, the DF value gradually increased at the UP/DP concentration ratio of 1:10 but decreased after the continuous increase of the UP/DP concentration ratio. The optimal UP/DP concentration ratio that resulted in a maximum DF value was 1:10 (Figure 2A). The FEN 1 drove the DNA walker-like reaction, and its reaction concentration should be optimized to obtain a highly efficient DNA walker-like reaction. As shown in Figure 2B, this increase in DF value with increasing FEN 1 concentration dominantly accounted for the FEN 1-driven DNA walker-like reaction. The results indicated that 10 U of FEN 1 contributed to the maximum DF value. An increase in reaction time could induce the WT-triggered DNA walker-like reaction and consequently affect the signal output of MT and WT (Figure 2C). The DF value showed a change, first increasing and then decreasing with the increment of reaction time, and reached the maximum DF value at 4 h. Therefore, 4 h was regarded as the optimal reaction time. Considering that the reaction temperature might affect the FEN 1-driven DNA walker-like reaction, the effect of the temperature on the DNA walker-like reaction was also investigated (Figure 2D). The DF value increased as the reaction temperature increased, and the maximum DF value was reached at 55°C, indicating that 55°C was the optimal reaction temperature.

### 3.4 Analytical performance of the biosensor

**FIGURE 3:**
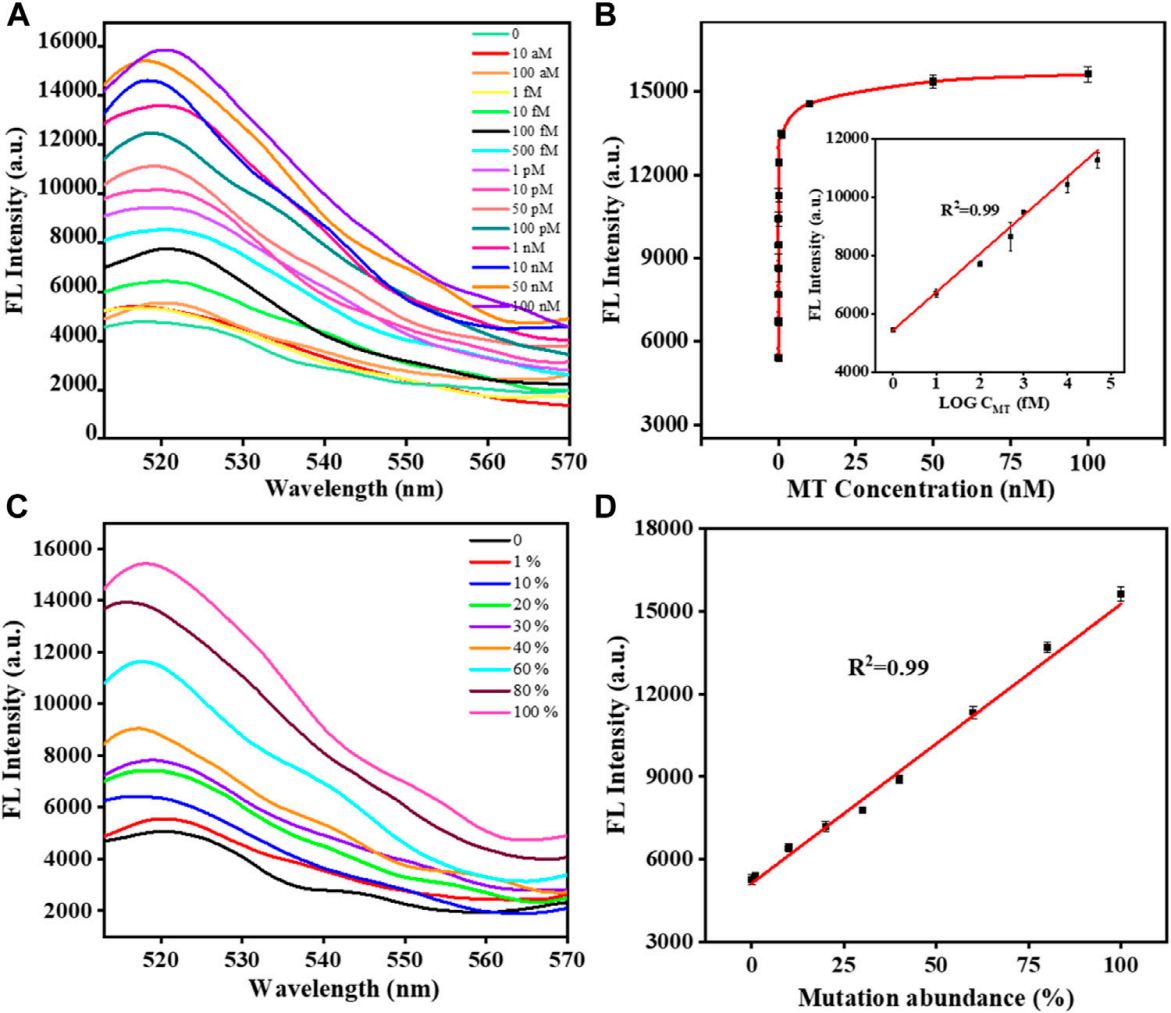
The fluorescence responses of the biosensor with different concentrations of MT **(A)**. The calibration plot of the fluorescence intensity *versus* the logarithm of the MT concentration **(B)**. The fluorescence responses of the biosensor with different abundances of MT **(C)**. The calibration plot of the fluorescence intensity *versus* the abundance of MT **(D)**. Error bars, SD, *n* = 3.

After optimizing the experimental parameters of the proposed biosensor, the analytical performance of the biosensor was further evaluated. In general, the fluorescence intensity gradually increased with the concentration of MT in the range of 1 fM-100 nM. A highly linear relationship was obtained between the fluorescence intensity and the logarithm of the MT concentration (Figures 3A, B). The equation derived from the linear regression was F = 1317.28×logC+5422.12 (R2 = 0.996). According to the 3σ/slope, the detection limit was 0.4 fM. The detection of different abundances of MT was important because MT coexists with WT in real samples. We tried to validate the ability to detect different abundances of MT in MT/WT mixtures in different proportions. As shown in Figures 3C, D, the fluorescence intensity was a linear response over a wide range of MT abundance from 0% to 100%. The linear equation was y = 102.16x+5231.98 (R2 = 0.995), where y was fluorescence intensity and x was MT abundance. It can be confirmed that this biosensor showed high selectivity and detection accuracy in KRAS gene mutant detection.

### 3.5 Selectivity and anti-interference of the biosensor

**FIGURE 4:**
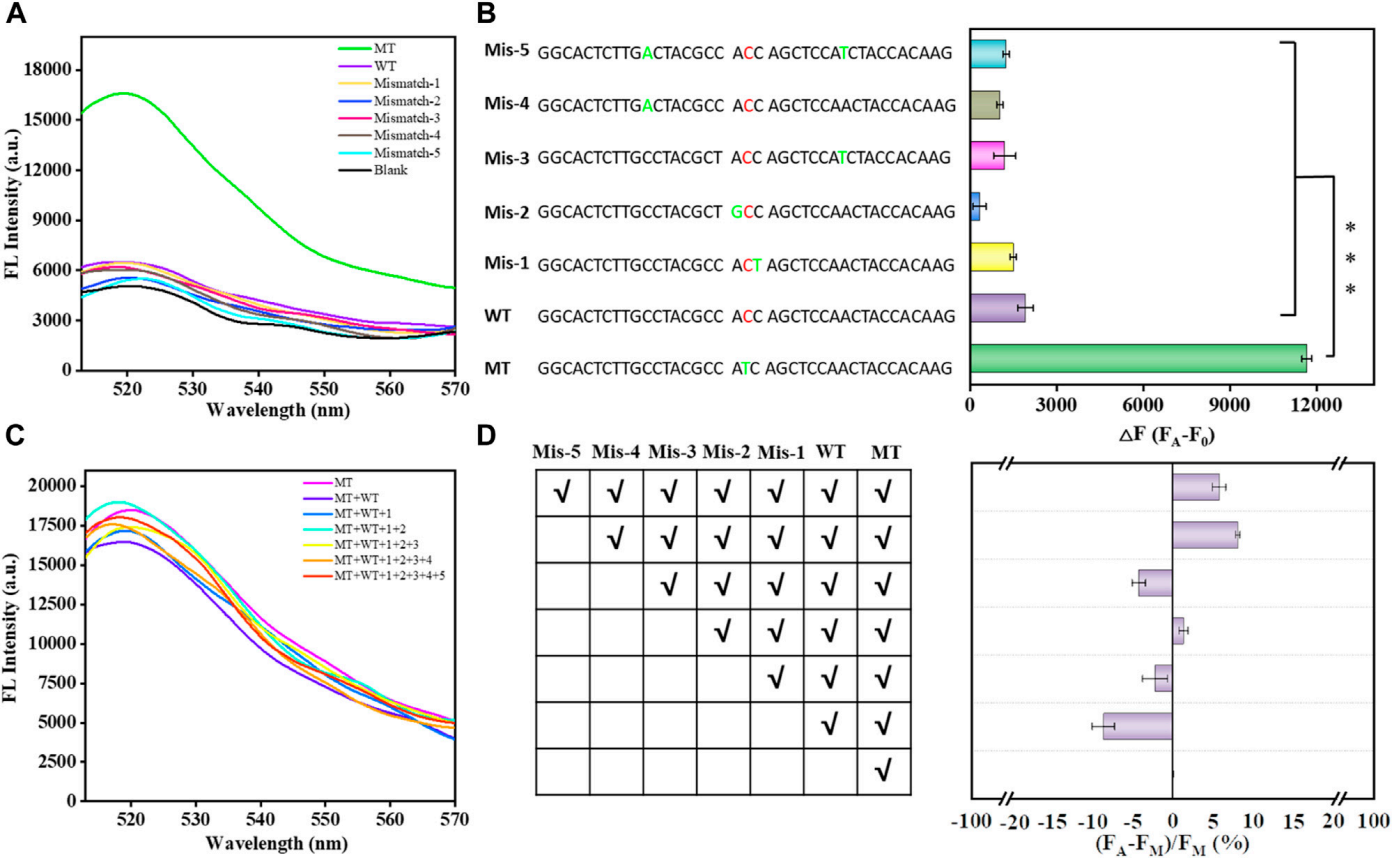
Selectivity of the proposed biosensor in analyzing single-base mismatched sequences and multiple-base mismatched sequences **(A)**. Bar graph reflecting the selectivity of the proposed biosensor in analyzing single base-mismatched sequences and multiple base-mismatched sequences **(B)**. Error bars, SD, *n* = 3. ***p < 0.001. Anti-interference of the proposed biosensor in the analysis of single-base mismatched sequences in a cumulative manner **(C)**. Bar graph reflecting the anti-interference of the proposed biosensor in the analysis of single-base mismatched sequences in a cumulative manner **(D)**.

According to the above results, the biosensor exhibited outstanding detection sensitivity. Next, we decided to verify its selectivity and anti-interference by using mismatched substances at different sites. As shown in Figures 4A, B, the biosensor could effectively discriminate between MT and single-base mismatched sequences at different sites; that is, the biosensor meets the selectivity in the real sample analysis. Moreover, the biosensor still exhibited high anti-interference to discriminate the single-base mismatched sequences in a cumulative manner (Figures 4C, D). The signal difference was less than 10% after the cumulative addition of mismatched sequences. Therefore, these results confirm that this biosensor can specifically and selectively detect the KRAS mutant gene in complex samples.

### 3.6 Real sample analysis

**FIGURE 5:**
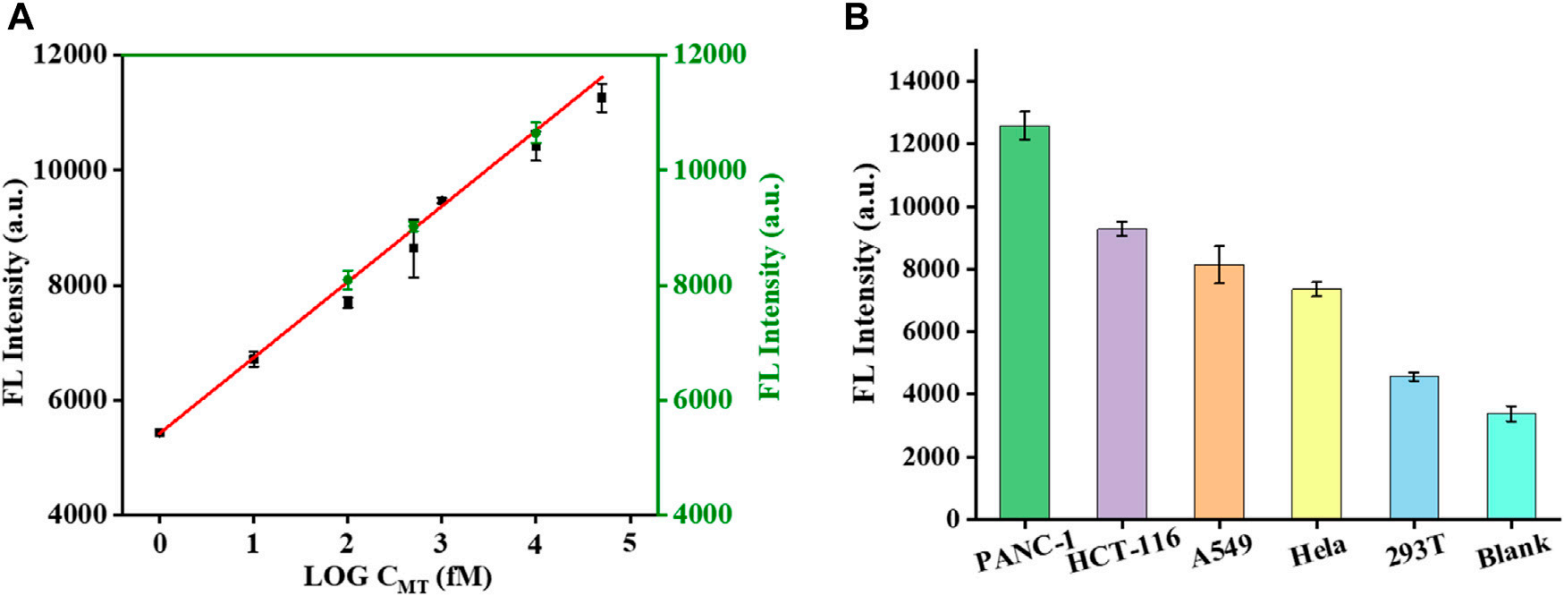
Effect of the specificity investigation on the real samples. Mutation expression levels of KRAS genes in different cell lines **(A)**. Deviation of test values after adding known concentrations of MT (0.1, 0.5, and 100 pM) to real samples **(B)**.

To further verify the accuracy of the KRAS mutant genes detected by the biosensor, the addition-recovery experiments were carried out by adding known concentrations of MT (0.1, 0.5, and 100 p.m.). The recoveries ranged from 104% to 106% (Figure 5A; Table 1). From Figure 5A, a small deviation of the sensor was observed compared with the simulated standard linear curve, indicating its excellent application performance. Next, we conducted performance tests on the sensor with real samples. We extracted DNA from PANC-1 cells, HCT-116 cells, A549 cells, Hela cells, and 293T cells and analyzed the KRAS mutant gene using this proposed biosensor. The fluorescence intensity represents the expression level of KRAS mutant genes in cancer cells. Interestingly, different cell types displayed different expression levels of KRAS mutant genes evaluated by this biosensor. We found that KRAS mutant genes had abundant expression in PANC-1 cells, while low expression was observed in 293 T cells (Figure 5B). These test results are consistent with those reported in the literature, indicating that our sensor is accurate (Sakamoto et al., 2022) and has certain application potential in real sample analysis.

**TABLE 1:** The recovery test of the proposed biosensor in 293 T cells (*n* = 3).

Sample	Added (pM)	Found (mean ± SD, pM)	Recovery (%)
1	0.1	0.106 ± 0.013	106
2	0.5	0.523 ± 0.044	104
3	100	105.8 ± 5.9	105.8

## 4 Conclusion

In summary, we have exploited a FEN 1-driven DNA walker-like reaction coupling with magnetic bead-based separation for specific SNP detection. The proposed biosensor has several outstanding advantages. First, DP was anchored to the SMB to increase the DP concentration, thereby accelerating the DNA walker-like reaction. Second, the SMB-based separation could enable a high separation of uncleaved DP to avoid the false-positive signal, thereby improving the specificity of the sensor. We have confirmed that this biosensor enabled specific and sensitive SNP detection, showing a linear response ranging from 1 fM to 100 nM with a detection limit of 0.4 fM. This sensing performance is superior to the previously reported methods shown in Supplementary Table S2 (Zhou et al., 2018; Wolfe et al., 2019; Song et al., 2021; Zhang et al., 2023a). Moreover, we also executed anti-interference and real sample analysis with this biosensor, which exhibited superior detection performance. The successful detection of SNP in real samples showed that the proposed biosensor has the potential to guide clinical use for the prediction, diagnosis, and treatment of a variety of diseases.

## Data Availability

The original contributions presented in the study are included in the article/Supplementary Material, further inquiries can be directed to the corresponding authors.
