# Supplementary material for: A FEN 1-driven DNA walker-like reaction coupling with magnetic bead-based separation for specific SNP detection
Source: Front Bioeng Biotechnol. 2023 Nov 3;11:1279473. doi: 10.3389/fbioe.2023.1279473 (PMC10656677; doi:10.3389/fbioe.2023.1279473)
Supplement: Supplementary file 1 [file Table1.docx]

Supplementary Material

# Table S1 The oligonucleotides used in the experiments

| Name | Sequence (5'-3') |
| --- | --- |
| Upstream prode | CTTGTGGTAGTTGGAGCTGT |
| Downstream prode | FAM-GCGTACAAGGTGCTCCATCTATGGCGTAGGCAAGAGTGCC TTTTTTTTTTTTTTTTTTTT-Bio |
| Mutant target | GGC ACT CTT GCC TAC GCC ATC AGC TCC AAC TAC CAC AAG |
| Wild-type target | GGC ACT CTT GCC TAC GCC ACC AGC TCC AAC TAC CAC AAG |
| Mismatch-1 | GGC ACT CTT GCC TAC GCC ACT AGC TCC AAC TAC CAC AAG |
| Mismatch-2 | GGC ACT CTT GCC TAC GCT GCC AGC TCC AAC TAC CAC AAG |
| Mismatch-3 | GGC ACT CTT GCC TAC GCT ACC AGC TCC ATC TAC CAC AAG |
| Mismatch-4 | GGC ACT CTT GAC TAC GCC ACC AGC TCC AAC TAC CAC AAG |
| Mismatch-5 | GGC ACT CTT GAC TAC GCC ACC AGC TCC ATC TAC CAC AAG |
| Blank | CGA CTA TTG CAC CAG CAT CGA GAG AGA CCA AAG TGACAT |

# Table S2 Comparison of SNP detection performance using different strategies.

| **Methods** | **Platforms** | **Detection limit** | **Reference** |
| --- | --- | --- | --- |
| Monitored colorimetrically using a pH indicator | Colorimetry | 11nM | ([Wolfe et al., 2019](#_ENREF_3)) |
| Nanosized germananes | Electrochemistry | 34 pM | ([Song et al., 2021](#_ENREF_2)) |
| Enzyme-free DNA template-directed click reaction for SNP detection | Laser-induced fluorescence | 25 fM | ([Zhou et al., 2018](#_ENREF_5)) |
| Enzyme-free and SNPs assay based on asymmetric MNAzyme probes | Fluorescence | 0.59 nM | ([Zhang et al., 2023](#_ENREF_4)) |
| DNA walker-like reaction coupling with magnetic bead-based | Fluorescence | 0.4 fM | The current study |

**References**

Song, Z., Ang, W.L., Sturala, J., Mazanek, V., Marvan, P., Sofer, Z., et al. (2021). Functionalized Germanene-Based Nanomaterials for the Detection of Single Nucleotide Polymorphism. *ACS Appl. Nano Mater.* 4(5)**,** 5164-5175. doi: 10.1021/acsanm.1c00606.

Wolfe, M.G., Ali, M.M., and Brennan, J.D. (2019). Enzymatic Litmus Test for Selective Colorimetric Detection of C-C Single Nucleotide Polymorphisms. *Anal. Chem.* 91(7)**,** 4735-4740. doi: 10.1021/acs.analchem.9b00235.

Zhang, X., Li, Q., Chao, Q., Zhang, Y., Sun, X., Fan, G.C., et al. (2023). A protein enzyme-free strategy for fluorescence detection of single nucleotide polymorphisms using asymmetric MNAzymes. *Anal Chim Acta* 1243**,** 340811. doi: 10.1016/j.aca.2023.340811.

Zhou, Q.Y., Yuan, F., Zhang, X.H., Zhou, Y.L., and Zhang, X.X. (2018). Simultaneous multiple single nucleotide polymorphism detection based on click chemistry combined with DNA-encoded probes. *Chem Sci* 9(13)**,** 3335-3340. doi: 10.1039/c8sc00307f.
